# Electrocardiographic abnormalities in centenarians: impact on survival

**DOI:** 10.1186/1471-2318-12-15

**Published:** 2012-04-20

**Authors:** Ramón Rabuñal-Rey, Rafael Monte-Secades, Adriana Gomez-Gigirey, Sonia Pértega-Díaz, Ana Testa-Fernández, Salvador Pita-Fernández, Emilio Casariego-Vales

**Affiliations:** 1 Internal Medicine Department, Lucus Augusti University Hospital (HULA), SERGAS, San Cibrao, s/n, 27003, Lugo, Spain; 2 Internal Medicine Department, Arquitecto Marcide Hospital, SERGAS, Av. da Residencia, s/n, 15405, Ferrol, Spain; 3 Clinic Epidemiology and Biostatistics Unit, A Coruña University Hospital (CHUAC), SERGAS, As Xubias, 15006, A Coruña, Spain; 4 Cardiology Department, Lucus Augusti University Hospital (HULA), SERGAS, San Cibrao, s/n, 27003, Lugo, Spain

## Abstract

**Background:**

The centenarian population is gradually increasing, so it is becoming more common to see centenarians in clinical practice. Electrocardiogram abnormalities in the elderly have been reported, but several methodological biases have been detected that limit the validity of their results. The aim of this study is to analyse the ECG abnormalities in a prospective study of the centenarian population and to assess their impact on survival.

**Method:**

We performed a domiciliary visit, where a medical history, an ECG and blood analysis were obtained. Barthel index (BI), cognitive mini-exam (CME) and Charlson index (ChI) were all determined. Patients were followed up by telephone up until their death.

**Results:**

A total of 80 centenarians were studied, 26 men and 64 women, mean age 100.8 (SD 1.3). Of these, 81% had been admitted to the hospital at least once in the past, 81.3% were taking drugs (mean 3.3, rank 0–11). ChI was 1.21 (SD 1.19). Men had higher scores both for BI (70 -SD 34.4- vs. 50.4 -SD 36.6-, *P* = .005) and CME (16.5 -SD 9.1- vs. 9.1 –SD 11.6-, *P* = .008); 40.3% of the centenarians had anaemia, 67.5% renal failure, 13% hyperglycaemia, 22.1% hypoalbuminaemia and 10.7% dyslipidaemia, without statistically significant differences regarding sex. Only 7% had a normal ECG; 21 (26.3%) had atrial fibrillation (AF), 30 (37.5%) conduction defects and 31 (38.8%) abnormalities suggestive of ischemia, without sex-related differences. A history of heart disease was significantly associated with the presence of AF (*P* = .002, OR 5.2, CI 95% 1.8 to 15.2) and changes suggestive of ischemia (*P* = .019, OR 3.2, CI 95% 1.2-8.7). Mean survival was 628 days (SD 578.5), median 481 days. Mortality risk was independently associated with the presence of AF (RR 2.0, *P* = .011), hyperglycaemia (RR 2.2, *P* = .032), hypoalbuminaemia (RR 3.5, *P* < .001) and functional dependence assessed by BI (RR 1.8, *P* = .024).

**Conclusion:**

Although ECG abnormalities are common in centenarians, they are not related to sex, functional capacity or cognitive impairment. The only abnormality that has an impact on survival is AF.

## Background

The centenarian population is gradually increasing, so it is becoming more common to see centenarians in clinical practice [1]. These patients have their own characteristics that are different compared to the "younger" elderly. Thus, a lower prevalence of diabetes mellitus [2], or an improved cardiovascular risk profile [3] in this group has been reported.

Electrocardiogram abnormalities (ECG) in the elderly are extensively reported [4-6]. They are frequent and arrhythmias and repolarisation abnormalities predominate. These studies mostly include population aged between 65 and 90 years old. By contrast, studies that analyse changes in the electrocardiogram in centenarians are rare. These studies report similar abnormalities but more often than those found in younger elderly. However, their sample size is low and several methodological biases have been detected (retrospective, no systematic assessments, etc.) that limit the validity of their results [7-14].

The aim of this study is to analyse the ECG abnormalities in a prospective study of the centenarian population in our health setting [15], their frequency, their relationship with sex and functional capacity and their value as a predictor of survival.

## Methods

Prospective follow-up observational study of 99-year-old and older patients living in the Lugo area (Galicia, northwest Spain), covering a population of 221,907 inhabitants. In January 2001, the information of all 99-year-old and older patients in this area was obtained from the National Health System register. After contact with the patients and/or their main caregiver, they were informed about the aims of the study and their consent to participate was requested. We conducted a domiciliary visit by a doctor and a nurse, who completed the study protocol, which included demographic data, medical history and physical examination. Barthel Index (BI) of basic activities of daily living [16], the cognition mini-exam (CME) (Spanish version of the Folstein Mini-mental State Exam) [17] and the Charlson Comorbidity Index (ChI) [18] were all determined. We performed a resting 12-lead ECG, and blood samples were obtained. Finally, when available, the hospital medical records were reviewed to confirm and complete the information obtained at home. Patients were monitored by telephone up until their death. The study was approved by the Galicia Clinical Research Ethics Committee.

The ECG was analysed independently by two researchers, according to the classification included in the Minnesota code [19]. Discrepancies were resolved by a second joint review of the ECG.

The following definitions were established: heart disease: clearly documented history of ischemic heart disease or heart failure; hypertension: systolic blood pressure > 140 mmHg or diastolic > 90 mmHg; functional dependence: BI score ≤60; cognitive impairment: CME score < 20; anaemia: haemoglobin < 13 g/L in men or < 12 g/L in women; renal failure: estimated glomerular filtration rate (MDDR-4) < 60 mL/min/1.73 m^2^; hyperglycaemia: fasting glucose > 126 mg/dL; hypoalbuminaemia: albumin < 3.5 g/dL; dyslipidaemia: total cholesterol > 220 mg/dL.

Statistical analysis: a descriptive study of the variables included in the study was performed. Quantitative variables were expressed as mean and standard deviation (SD). Qualitative variables were expressed as absolute value and percentage. In the univariate analysis we performed the comparison of numerical parameters between test groups using the student *t* test or Mann Whitney test, as appropriate, after verification of normality using the Kolmogorov-Smirnov test. For the comparison of qualitative variables, the chi square test was performed, Fisher´s exact test was used when the cells contained expected values less than five. Kaplan-Meier analysis was used to study survival, comparing survival between groups by the log-rank test. To study the combined effect of several variables in the forecast, we used a Cox regression model, considering the maximum model to be those variables statistically significant in the univariate analysis. Statistical significance was set at *P* < .05. Statistical analysis was performed using SPSS 17.0 for Windows.

## Results

Of a total population of 84 centenarians, 80 (95.2%) agreed to participate in the study, 26 men and 64 women, mean age of 100.8 (SD 1.3). Table 1 shows the clinical features of the series. Only nine patients did not have a prior diagnosis of cardiovascular disease, dementia or cancer and had a good functional status.

**Table 1:** Clinical variables of centenarians in the series according to sex

	**Total**			**Male**			**Female**			***P***
	**No.**	**Mean**	**SD**	**No.**	**Mean**	**SD**	**No.**	**Mean**	**SD**	
**BMI**	71	24.1	4.3	23	24.8	3.8	48	23.8	4.5	.388
**BPs**	77	131.7	21.2	24	139.3	26.0	53	128.3	17.9	.223
**BPd**	77	73.0	12.7	24	74.4	14.5	53	72.4	11.9	.411
**HR**	80	77.0	14.5	26	74.1	11.8	54	78.3	15.5	.224
	**No.**	**%**		**No.**	**%**		**No.**	**%**		
**Smoker**	17	21.3%		17	65.4%		0	0%		.000
**Diabetes mellitus**	9	11.3%		3	11.5%		6	11.1%		.999
**Hypertension**	21	26.3%		3	11.5%		18	33.3%		.056
**Dyslipaemia**	3	3.8%		2	7.7%		1	1.9%		.245
**CVD**	24	30.0%		5	19.2%		19	35.2%		.145
**Dementia**	26	32.5%		5	19.2%		21	38.9%		.079
**Prior hospital admission**	65	81.3%		23	88.5%		42	77.8%		.363
Drugs*	65	81.3%		19	73.1%		46	85.2%		.194
	**No.**	**%**		**No.**	**%**		**No.**	**%**	**OR (CI)**	
**Charlson index score ≥ 2**	26	32.5%		9	34.6%		17	31.5%	0.9 (0.3-2.3)	.779
**Barthek index score ≤ 60**	37	46.3%		8	30.8%		29	53.7%	0.4 (0.1-1.03)	.054
**Cognition miniexam score < 20**	57	71.3%		12	46.2%		45	83.3%	0.2 (2.0-16.7)	.001
**Anaemia**	31	40.3%		13	50%		18	35.3%	0.5 (0.2-1.4)	.230
**Hyperglycaemia**	10	13%		2	7.7		8	15.7	2.2 (0.4-11.4)	.480
**Renal failure**	52	67.5%		14	53.8%		38	74.5%	2.5 (0.9-6.8)	.067
**Dyslipidaemia**	8	10.7%		4	16.0%		4	8.0%	0.5 (0.1-2.0)	.429
**Hypoalbuminaemia**	17	22.1%		7	26.9%		10	19.6%	0.7 (0.2-2.0)	.464

*mean 3.2 (SD 2.1) drugs per patient (rank 0–11).

Abbreviations: BPd: diastolic blood pressure; BPs: systolic blood pressure. BMI: body mass index; CI: 95% confidence interval; CVD: cardiovascular disease; HR: heart rate; OR: Odds ratio; SD: standard deviation.

Anaemia: haemoglobin < 12 g/dL in women, <13 g/dL in men. Hyperglycaemia: fasting glycaemia > 126 mg/dL. Renal failure: glomerular filtration rate (MDDR-4) < 60. Dyslipidaemia: total cholesterol > 220 mg/dL. Hypoalbuminaemia: albumin < 3.5 g/dL.

Only seven patients (8%) had a completely normal ECG. No cases of atrial flutter were registered. Atrial fibrillation (AF) was found in 21 patients (26.3%), AV conduction defect in nine (11.3%), ventricular conduction disturbance in 26 (32.5%), and changes suggestive of ischaemia (abnormal repolarisation patterns or Q/QS) in 31 patients (38.8%). No statistically significant differences were found for any of the parameters studied regarding sex (Table 2).

**Table 2:** Distribution of abnormalities in the ECG of 80 centenarians, classified by the Minnesota code

	**Total n = 80**		**Male n = 26**		**Female n = 64**		***P***
	**No.**	**%**	**No.**	**%**	**No.**	**%**	
**Sinus rhythm**	59	73.8%	19	73.1%	40	74.1%	.924
**Arrhythmias**	44	55%	15	57.7%	29	53.7%	.737
Premature supraventricular, junctional or ventricular beats	29	36.3%	8	30.8%	21	38.9%	.479
Atrial fibrillation	21	26.3%	7	26.9%	14	25.9%	.924
Other arrhythmias	5	6.3%	2	7.7%	3	5.6%	.658
**QRS axis deviation**	27	33.8%	9	34.6%	18	33.3%	.910
Left-axis deviation	24	30%	7	26.9%	17	31.5%	.677
Right-axis deviation	1	1.3%	0	0%	1	1.9%	.999
Extreme axis deviation	2	2.5%	2	7.7%	0	0%	.103
**High amplitude R waves**	7	8.8%	1	3.8%	6	11.1%	.418
Left ventricular hypertrophy	7	8.8%	1	3.8%	6	11.1%	.418
**AV conduction defect**	9	11.3%	3	11.5%	6	11.1%	.999
Second-degree AV block	1	1.3%	0	0%	1	1.9%	.999
First-degree AV block	8	10%	3	11.5%	5	9.3%	.710
**Ventricular conduction defect**	26	32.5%	11	42.3%	15	27.8%	.194
Left bundle branch block	8	10%	1	3.8%	7	13%	.264
Right bundle branch block	12	15%	6	23.1%	6	11.1%	.160
Right bundle branch block incomplete	2	2.5%	0	0%	2	3.7%	.999
Left bundle branch block incomplete	7	8.8%	4	15.4%	3	5.6%	.206
Nonspecific intraventricular conduction delay	2	2.5%	2	7.7%	0	0%	.103
**Repolarisation abnormalities**	25	31.3%	8	30.8%	17	31.5%	.949
ST depression	17	21.3%	3	11.5%	14	25.9%	.242
T amplitude zero, negative or diphasic	18	22.5%	7	26.9%	11	20.4%	.511
Other repolarisation abnormalities	5	6.3%	2	7.7%	3	5.6%	.658
**Q and QS patterns**	13	16.3%	5	19.2%	8	14.8%	.616
**Miscellaneous items**	27	33.8%	6	23.1%	21	38.9%	.161
Low QRS amplitude	9	11.3%	3	11.5%	6	11.1%	.999
Atrial enlargement	7	8.8%	1	3.8%	6	11.1%	.418
QRS transition zone to the right/left	15	18.8%	3	11.5%	12	22.2%	.363

A history of heart disease was significantly associated with the presence of AF (*P* = .002, OR 5.2, 95% CI 1.8 to 15.2) and with changes suggestive of ischemia (*P* = .019, OR 3.2, 95% CI 1.2-8.7), both ST segment depression (*P* = .003) and a Q/QS pattern (*P* = .007).

Dependent patients for activities of daily living (BI ≤ 60) showed, compared to independent patients, a higher frequency of AF (32.4% vs. 20.9%), abnormal axis (40.5% vs. 27.9% ), AV conduction defects (16.2% vs. 7%) and repolarisation abnormalities (37.8% vs. 25.6%), but statistically significant differences were not found. ECG changes suggesting ischemic disease (ST depression or Q/QS pattern) or conduction defects were more common in patients with cognitive impairment, but differences were not statistically significant. Table 3 shows the differences between some ECG abnormalities according to the presence of cognitive impairment and functional dependence.

**Table 3:** Distribution of main abnormalities in the ECG of 80 centenarians, according to the presence of functional dependence measured by BI and cognitive impairment measured by CME

	**BI > 60**		**BI ≤ 60**		***P***	**CME ≥ 20**		**CME < 20**		***P***
	**No.**	**%**	**No.**	**%**		**No.**	**%**	**No.**	**%**	
**Sinus rhythm**	34	79.1	25	67.6	.244	17	73.9%	42	73.7	.983
**Arrhythmias**	24	55.8	20	54.1	.875	14	60.9%	30	52.6%	.503
Atrial fibrillation or flutter	9	20.9	12	32.4	.244	6	26.1%	15	26.3%	.983
**QRS axis deviation**	12	27.9	15	40.5	.233	6	26.1%	21	36.8%	.357
**High amplitude R waves**	6	14	1	2.7	.116	2	8.7%	5	8.8%	.999
**AV conduction defect**	3	7	6	16.2	.290	2	8.7%	7	12.3%	.999
**Ventricular conduction defect**	14	32.6	12	32.4	.990	6	26.1%	20	35.1%	.437
**Repolarisation abnormalities**	11	25.6	14	37.8	.238	5	21.7%	20	35.1%	.244
ST depression	6	14	11	29.7	.085	2	8.7%	15	26.3%	.130
T amplitude zero, negative or diphasic	8	18.6	10	27	.368	4	17.4%	14	24.6%	.487
**Q and QS patterns**	5	11.6	8	21.6	.227	3	13.0%	10	17.5%	.747
**Miscellaneous items**	13	30.2	14	37.8	.473	4	17.4%	23	40.4%	.068

Abbreviations: BI, Barthel index; CME, cognition mini-exam.

**Figure 1:**
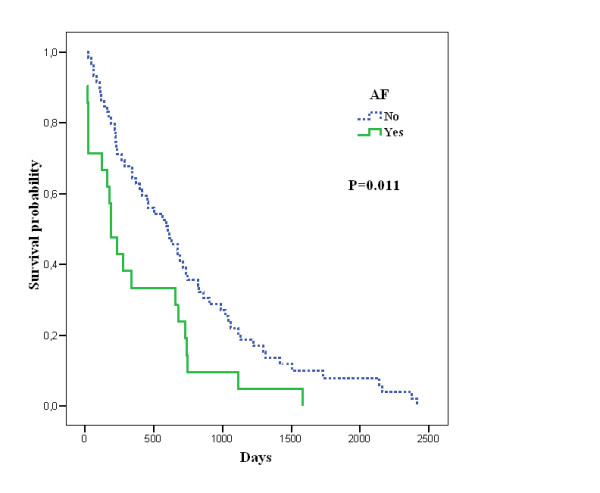
Kaplan-Meier survival curve in days according to presence of AF.

Mean survival was 628 days (SD 578.5), median 481 days, with no statistical differences between sexes. Table 4 shows survival in days according to the presence or absence of abnormalities in the basal ECG. In the univariate analysis, among the abnormalities in the basal ECG, those significantly associated with lower survival were AF (median survival: 191 vs. 603 days, *P* = .011) (Figure 1) and ST depression (median survival: 191 vs. 603 days, *P* = .004).

**Table 4:** Univariate analysis of electrocardiographic abnormalities associated with survival

		**n**	Median*	**RR**	**95% CI RR**	***P***
**Atrial fibrillation**	yes	21	191	1.9	1.2 - 3.2	***.012***
	no	59	603			
**QRS axis deviation**	yes	27	368	1.0	0.6 - 1.6	.958
	no	53	627			
**High amplitude R waves**	yes	7	266	1.8	0.8 - 4	.136
	no	73	564			
**AV conduction defect**	yes	9	395	1.5	0.8 – 3.1	.231
	no	71	590			
**Ventricular conduction defect**	yes	26	459	0.8	0.5 – 1.3	.417
	no	54	454			
**Repolarisation abnormalities**	yes	25	266	1.4	0.9 – 2.2	.192
	no	55	590			
ST depression	yes	17	191	2.2	1.3 – 3.9	***.005***
	no	63	603			
**Q and QS patterns**	yes	13	340	1.4	0.8 – 2.6	.275
	no	67	564			
**Miscellaneous items**	yes	27	344	1.4	0.8 – 2.2	.199
	no	53	627			

*Survival days.

Abbreviations: CI 95%: 95% Confidence interval; RR: Relative risk.

A logistic regression model was applied for multivariate analysis. In obtaining the maximum model, the factors that proved significant in the univariate analysis were used as well as those variables thought to possibly influence final results: age, sex, history of cardiovascular disease, stroke o dementia, BMI < 20, anaemia, leukocytosis, hyperglycaemia, hypoalbuminaemia, increased ferritin, dyslipidaemia, atrial fibrillation, ST depression, comorbidity, functional dependence and cognitive impairment.

After adjustment for age, sex, diabetes, dyslipidaemia, albumin and functional dependence, the presence of AF was significantly associated with mortality risk (RR 2.0, *P* = .011). Other variables associated with a worse prognosis were hyperglycaemia (RR 2.2, *P* = .032), anaemia (RR 3.5, *P* < .001) and functional dependence (RR 0.6, *P* = .024) (Table 5).

**Table 5:** Multivariate analysis of factors associated with mortality

	**β**	***P***	**RR**	**95% CI RR**
Sex (male)	0.2	.561	1.2	0.7 - 2.0
Age (years)	−0.2	.154	0.8	0.7- 1.1
Functional dependence	0.6	.024	1.8	1.1 - 2.9
Hyperglycaemia	0.8	.032	2.2	1.1 - 4.6
Hypoalbuminaemia	1.2	.000	3.5	1.9 - 6.5
Atrial fibrillation	0.7	.011	2.0	1.2 - 3.5

Hypoalbuminaemia: Albumin < 3.5 g/dL.

Hyperglycaemia: Fasting glycaemia > 126 mg/dL.

Functional dependence: Barthel index ≤ 60.

RR: Relative risk. CI 95%: 95% Confidence interval.

## Discussion

In our study only 8% of centenarians have a completely normal ECG. However, the abnormalities most frequently found have little clinical relevance, they are not related to their functional capacity, and only AF is associated with increased mortality.

The ECG abnormalities that appear with age have been widely studied. The most common findings are left ventricular hypertrophy, repolarisation abnormalities and Q/QS patterns, even in asymptomatic patients [4,6]. The frequency of these findings increases with age [4,5], and in some studies are associated with increased mortality [20,21]. In general, men have more abnormalities related to ischemic heart disease [4,6], although this difference fades with age [5]. Among the rhythm disturbances, the most common are extra systolic beats, followed by AF [4-6,22] and their prevalence also increases with age. Other minor abnormalities are commonly found, so that less than 40% of those over 65 have a completely normal ECG.

By contrast, ECG studies in centenarians are rare and their results have a wide variability [6-14]. For example, the frequency of AF varies from 0 to 30%, or the ST segment changes from 9% to 40%. This is probably because of the difficulty of obtaining a wide sample [23], which introduces many biases: small numbers of patients [7,8,10,13], studies without established inclusion criteria, selected population collected [8,13] or retrospective studies in a hospital setting [14]. In addition, some of them do not perform a systematic analysis according to established criteria, such as the Minnesota code [9,11,12]. In most studies the analysis is purely descriptive and differences by sex, cardiovascular disease or functional capacity are not considered. Similarly, they do not discuss whether these findings are associated with a higher mortality in centenarians, as in a younger population [24].

Our study was designed to avoid these biases. We included the 95.2% of centenarians in our area, a systematic approach to baseline health status was performed at the patient's home, and they were followed up to their death. The main limitation that arises is the relatively low number of patients finally included, 80. Nonetheless, we consider that the sample is large enough compared to previous studies on centenarians.

Only 8% of centenarians had a normal ECG; thus, the frequency of ECG alterations was very high. This finding has been previously reported in an older population [5] and also in centenarians [13,14]. The fact that the frequency of ECG abnormalities increases with age, together with the absence of differences according to sex in our series, suggest that these abnormalities are related to aging itself more than to cardiovascular risk factors, which are more prevalent in men. Our data revealed that these changes were not related to greater disability or to cognitive impairment (Table 3), probably because they are associated with a wide range of factors, of which heart disease is one of several.

Arrhythmias are common in these patients (55%) with extrasystole the most frequent. Interestingly, the frequency of AF in our series was high (26%), whereas such a prevalence has only been reported in a hospital-based retrospective study [14]. Nevertheless, these data are consistent with those obtained in the general population over 85 [5]. The lower prevalence of AF observed in previous studies on centenarians [8-13] is probably related to the aforementioned sample bias.

The presence of left axis in 33.8% of cases, and left ventricular hypertrophy in 8.8% are both within the range reported in previous studies [7,8,13]. The frequency of AV or intraventricular conduction defects was also high, similar to that reported in the centenarian population, and clearly superior to that reported in non-centenarian elderly [4]. This is probably because of the aging process, also affecting the conduction tissue. Changes suggestive of ischaemia (disorder of repolarisation 31.3%, Q/QS pattern 16.3%), were also more frequent than those observed in a younger population, but not in the same proportion. This fact might suggest a bias of survival in the elderly who have no ischaemic heart disease.

In our series, AF has proven to be an independent predictor of mortality, a fact already reported in younger patients [21]. This situation probably reflects a more serious underlying heart disease [22]. This suggests that centenarians with AF could benefit from strict clinical control and optimal pharmacological treatment. In the same way, preventive strategies such as anticoagulation, which have shown a proven benefit in the younger population, might be considered in functionally independent centenarians without cognitive impairment.

## Conclusion

Although ECG abnormalities are common in centenarians, they are not related to sex, functional capacity or cognitive impairment. Most of them have little clinical or prognostic significance. These abnormalities seem to be the result of the natural aging process and the only one that had an impact on survival was AF.

## Competing interests

The authors declare that they have no competing interests.

## Authors’ contributions

RRR conceived the study, analyzed and interpreted the data and was a major contributor in writing the manuscript. RMS analyzed and interpreted the data and was a major contributor in writing the manuscript. AGG has analyzed the data and has been involved in drafting the manuscript and revising it critically. SPD participated in the design of the study and performed the statistical analysis. ATF has analyzed the data and has been involved in revising the manuscript critically. SPF participated in the design of the study and has been involved in revising the manuscript critically. ECV has analyzed the data and has been involved in drafting the manuscript and revising it critically. All authors approved the final manuscript.

## Pre-publication history

The pre-publication history for this paper can be accessed here:

http://www.biomedcentral.com/1471-2318/12/15/prepub

## References

[B1] Rabuñal Rey R Monte Secades R Rigueiro Veloso MT Casariego Vales EJ Ibáñez Alonso MD García Pais MJ Centenarian patients attended at a general hospital Rev Clin Esp 2002 202 326 8 12093397 10.1016/s0014-2565(02)71067-9

[B2] Motta M Bennati E Capri M Ferlito L Malaguarnera M Diabetes mellitus in the extreme longevity Exp Gerontol 2008 43 102 5 10.1016/j.exger.2007.06.012 17689906

[B3] Bennati E Murphy A Cambien F Whitehead AS Archbold GP Young IS Rea IM BELFAST centenarians: a case of optimised cardiovascular risk? Curr Pharm Des 2010 16 789 95 10.2174/138161210790883697 20388089

[B4] Campbell A Caird FI Jackson TF Prevalence of abnormalities of electrocardiogram in old people Br Heart J 1974 36 1005 11 10.1136/hrt.36.10.1005 4279682 PMC1020051

[B5] Rajala S Kaltiala K Haavisto M Mattila K Prevalence of ECG findings in very old people Eur Heart J 1984 5 168 74 6723687 10.1093/oxfordjournals.eurheartj.a061627

[B6] Furberg CD Manolio TA Psaty BM Bild DE Borhani NO Newman A Tabatznik B Rautaharju PM Major electrocardiographic abnormalities in persons aged 65 years and older (the Cardiovascular Health Study). Cardiovascular Health Study Collaborative Research Group Am J Cardiol 1992 69 1329 35 10.1016/0002-9149(92)91231-R 1585868

[B7] Masuzaki S Niimura T Tanaka H Nagoshi T Kashima T Ishigami T Tanaka N Kanehisa T Analysis of electrocardiogram of centenarians. Proceedings of the 33 rd Annual Meeting of the Japanese Circulation Society Jpn Circ J 1969 33 1158 9

[B8] Cornu JF Electrocardiogram of centenarians (apropos of 25 ECGs of centenarians) Rev Med Suisse Romande 1979 99 107 13 441617

[B9] Rohla M Lengyel E Beregui E Studies of centenarians in Hungary: results of twelve–lead electrocardiografic analysis Centenarians in Hungary. A sociomedical and demographic study 1990 27 Interdiscipl Top Gerontol, Basel, Karger 47 52

[B10] Wakida Y Okamoto Y Iwa T Yonemoto T Kanemaki K Shiomi T Mizutani K Kobayashi T Arrhythmias in centenarians Pacing Clin Electrophysiol 1994 17 11 Pt 2 2217 21 7845846 10.1111/j.1540-8159.1994.tb03829.x

[B11] Chessari S Mangiacavallo G Pollina R Priola P Pupella E Di Gregoli A Fradá G Twelve-lead electrocardiographic analysis and pharmacotherapy in centenarians Arch Gerontol Geriatr 1996 22 Suppl 1 367 72 18653058 10.1016/0167-4943(96)86963-9

[B12] Suzuki M Wilcox BJ Wilcox CD Implications from and for food cultures for cardiovascular disease: longevity Asia Pac J Clin Nutr 2001 10 165 71 10.1046/j.1440-6047.2001.00219.x 11710359

[B13] Klich-Raczka A Zyczkowska J Grodzicki T Electrocardiogram in centenarians Kardiol Pol 2003 58 275 81 14517559

[B14] Lakkireddy DR Clark RA Mohiuddin SM Electrocardiographic findings in patients >100 years of age without clinical evidence of cardiac disease Am J Cardiol 2003 92 1249 51 10.1016/j.amjcard.2003.07.046 14609614

[B15] Rabuñal Rey R Monte Secades R Veiga Cando MD Rigueiro Veloso MT López Díaz MJ Casariego Vales EJ Guerrero Lombardía J Health status of the oldest old: functional and medical situation in centenarians An Med Interna 2004 21 543 7 15538904 10.4321/s0212-71992004001100005

[B16] Cid-Ruzafa J Damián-Moreno J Disability evaluation: Barthel's index Rev Esp Salud Publica 1997 71 127 37 10.1590/S1135-57271997000200004 9546856

[B17] Lobo A Saz P Marcos G Día JL de la Cámara C Ventura T Revalidation and standardization of the cognition mini-exam (first Spanish version of the Mini-Mental Status Examination) in the general geriatric population Med Clin (Barc) 1999 112 767 74 10422057

[B18] Charlson ME Pompei P Ales KL MacKenzie CR A new method of classifying prognostic comorbidity in longitudinal studies: development and validation J Chronic Dis 1987 40 373 83 10.1016/0021-9681(87)90171-8 3558716

[B19] Rose GA Blackburn H Cardiovascular survey methods Monogr Ser World Health Organ 1968 56 1 188 4972212

[B20] Caird FI Campbell A Jackson TF Significance of abnormalities of electrocardiogram in old people Br Heart J 1974 36 1012 8 10.1136/hrt.36.10.1012 4279683 PMC1020052

[B21] Rajala S Haavisto M Kaltiala K Mattila K ECG findings and survival in very old people Eur Heart J 1985 6 247 52 4029181 10.1093/oxfordjournals.eurheartj.a061848

[B22] Furberg CD Psaty BM Manolio TA Gardin JM Smith VE Rautaharju PM Prevalence of atrial fibrillation in elderly subjects (the Cardiovascular Health Study) Am J Cardiol 1994 74 236 41 10.1016/0002-9149(94)90363-8 8037127

[B23] Andersen-Ranberg K Schroll M Jeune B Healthy centenarians do not exist, but autonomous centenarians do: a population-based study of morbidity among Danish centenarians J Am Geriatr Soc 2001 49 900 8 10.1046/j.1532-5415.2001.49180.x 11527481

[B24] Casiglia E Spolaore P Ginocchio G Marchioro M Mazza A di Menza G Maniati G Daskalakis C Colangeli G Ambrosio GB Mortality in relation to Minnesota code items in elderly subjects. Sex-related differences in a cardiovascular study in the elderly Jpn Heart J 1993 34 567 77 10.1536/ihj.34.567 8301843

